# Taking chances and making mistakes: non-genetic phenotypic heterogeneity and its consequences for surviving in dynamic environments

**DOI:** 10.1098/rsif.2017.0141

**Published:** 2017-07-12

**Authors:** Coco van Boxtel, Johan H. van Heerden, Niclas Nordholt, Phillipp Schmidt, Frank J. Bruggeman

**Affiliations:** Systems Bioinformatics, Amsterdam Institute for Molecules, Medicines and Systems (AIMMS), VU Amsterdam, De Boelelaan 1085, 1081 HV Amsterdam, The Netherlands

**Keywords:** phenotypic diversification, phenotypic adaptation, natural selection, bet-hedging, nutrient transitions

## Abstract

Natural selection has shaped the strategies for survival and growth of microorganisms. The success of microorganisms depends not only on slow evolutionary tuning but also on the ability to adapt to unpredictable changes in their environment. In principle, adaptive strategies range from purely deterministic mechanisms to those that exploit the randomness intrinsic to many cellular and molecular processes. Depending on the environment and selective pressures, particular strategies can lie somewhere along this continuum. In recent years, non-genetic cell-to-cell differences have received a lot of attention, not least because of their potential impact on the ability of microbial populations to survive in dynamic environments. Using several examples, we describe the origins of spontaneous and induced mechanisms of phenotypic adaptation. We identify some of the commonalities of these examples and consider the potential role of chance and constraints in microbial phenotypic adaptation.

## 1. Microbial biodiversity and phenotypic plasticity: one of life's many marvels

Microorganisms occupy an enormous number of niches on Earth; they are its most abundant life form. This evolutionarily success points to the remarkable flexibility and adaptability of microorganisms, not the least because their niches vary greatly. Although niches can be stable on a long time scale, many of them are characterized by highly dynamic conditions, with frequent fluctuations in environmental variables (e.g. nutrients, temperature, osmolarity etc.). Microbes are therefore forced to continuously adapt their phenotype to changing conditions, to survive and to prevent being out-competed by other species or genetic variants. The mechanisms for phenotypic adaptation are continuously tinkered by evolution, via mutation and selection. Their variety underscores the intriguing resourcefulness of microbe subpopulations in coping with environmental dynamics. Adaptation to new niches and sustaining their occupancy, therefore, relies on phenotypic adaptation, on a short time scale, and its slow evolutionary tuning, via mutations on a longer time scale [1].

Our view of microorganisms, and in particular of their amazing phenotypic plasticity, is also still evolving. The classical view, which emphasizes the determinacy of the phenotype from the genotype and the environment, has in the past decade been challenged by observations of partial indeterminacy, as underscored by phenotypic heterogeneity [2,3]. Single-cell studies invariably indicate that the molecular state varies between isogenic cells, even at constant conditions for sister cells, sharing the same mother cell [4]. This heterogeneity is caused by various stochastic phenomena in a cell, which can even lead to the emergence of subpopulations of cells with qualitatively different phenotypes, known as phenotypic diversification [5]. Population diversification can be a potent fitness enhancer for a population of microorganisms. For instance, to survive sudden extinction-threatening conditions, bacteria can enter a dormant, resilient physiological state to become a ‘persister cell’ [6]. Stochastic phenomena can also be fitness-reducing. They can distort information transmission and perturb regulatory mechanisms in cells to such an extent that adaptation dynamics to a new state is affected, possibly leading to maladaptation [7].

Phenotypic heterogeneity indicates that an understanding of microbial phenotypic adaptation requires studies of single cells. Inevitable molecular stochasticity can cause isogenic cells to adapt differently. The precise state that a cell is in, when conditions change, therefore determines its adaptation dynamics; whether it successfully adapts or not, and, if it does, how long this adaptation takes [8]. This is probably even more pronounced for eukaryotic microorganisms. Their phenotype is cell-cycle-stage dependent, which constitutes an additional ‘deterministic’ factor of phenotypic variability [9]. Cell-to-cell differences in adaptation dynamics force us to revisit ‘understood’ classical environmental transitions studies, such as nutrient transitions, which were mostly population-based, and take into account the impact of molecular stochasticity [7,8,10,11]. Below, we discuss how this new paradigm has led to surprising insights and novel systemic relations between cellular growth and stress tasks.

Eludicating how the stochasticity of specific molecular circuits influences fitness is not a simple task. This is perhaps surprising given the simplicity of the definition of microbial (geometric) fitness; which is the factor of increase in the number of viable offspring during some period of particular (dynamic) environmental conditions [12–14]. The complication arises from the fact that phenotypic heterogeneity in the context of fitness is still poorly understood and difficult to quantify, with many open questions that are hard to answer. Is phenotypic heterogeneity an evolved trait or is it the inevitable consequence of physico-chemical constraints and limitations in molecular circuits? In other words, how much of the cell-to-cell variability we observe in phenotypic traits arises from selection of noise-generating mechanisms (e.g. because it enhances fitness under certain conditions) and how much of it is due simply to physico-chemical limits in molecular circuits that cannot easily be improved by evolution (because it will result in a trade-off)? What are the selective pressures that promote heterogeneity? To what extent are the fitness consequences of phenotypic heterogeneity dependent on time scales and subpopulation sizes? First efforts to answer these questions have been undertaken [12,15–18], but it remains a challenge for modern biology to expand on them and finally give definite answers.

Natural selection enhances the occurrence of microorganisms with phenotypic adaptation mechanisms, including those that generate stochasticity, provided that they confer a fitness advantage. Such mechanisms may involve different types of molecular circuits that contribute to fitness such as signalling, metabolism, motility and stress. So even though fitness itself is one-dimensional (it is a single number), a cell sets this number via a multidimensional mechanism, which resembles a ‘single-objective, multi-task optimization’ problem. Similar problems occur in other disciplines such as in control engineering and finance. The resemblance is even deeper; the theories used in evolution to understand the fitness of organisms in dynamic environments have many similarities with theories used in other disciplines [12,13,17,19].

A successful fitness theory allows for descriptions of phenotypic adaptation ‘strategies’ at various levels of abstraction; from coarse and phenomenological descriptions to detailed molecular-mechanistic models [12,17,20]. Such a theory allows for the evaluation of adaptive strategies in terms of the fitness benefits of different molecular circuits and their fitness costs, associated with their consumption of limited biosynthetic resources [21,22] and inevitable stochastic disturbances. In our opinion, such an integrative, systemic view is ultimately required to understand the phenotypic adaptation of a bacterial species. It appears that, with existing fitness theories and experimental capabilities, this can indeed be achieved [13,15–20,23,24]. Much development is still required to achieve a comprehensive understanding of phenotypic adaptation, which we shall return to in the closing section of this review.

In this review, we address several aspects of the role of stochasticity in phenotypic adaptation by microorganisms; i.e. how it influences fitness, given the adaptational challenges that microbes face in their dynamic environments. We focus on phenotypic adaptation, hence on the process of a cell with a fixed genome that is changing its physiological behaviour. We will discuss several cases of phenotypic heterogeneity. In some examples, the fitness consequences are evident while for others they are more speculative. We aim to describe many of the possible roles of non-genetic heterogeneity in the adaptation strategies of microorganisms in dynamic environments.

## 2. Pioneering single-cell work

Nowadays, we exploit fluorescence microscopes and fluorescent reporters to study the surprising behaviours of single cells. This was not possible decades ago. However, already in the 1950s many researchers started asking questions about the functioning of single cells. The questions they asked are very similar to those that are most pressing now. For instance, they realized that the behaviour of individual cells in isogenic populations could deviate from the population average. The existence of subpopulations, that they likely form via chance events, that single cells vary in molecule copy numbers and show variable birth and division length, instantaneous growth rates and generation times were all being considered experimentally and theoretically [25–33]. How we study single cells now, using fluorescence microscopy and fluorescent reporters [34], saw an enormous growth after the introduction of several influential papers in the early 2000s [35–38]. Then, the focus was mostly on noise of molecular circuits, without much consideration of the cellular effects of molecular chance events. Recent work is mostly dealing with how systemic behaviour with a fitness effect varies from cell to cell. This review will be mostly concerned with the latter work.

## 3. Individuality in the responses of isogenic cells to nutrient transitions

**Figure 1.**
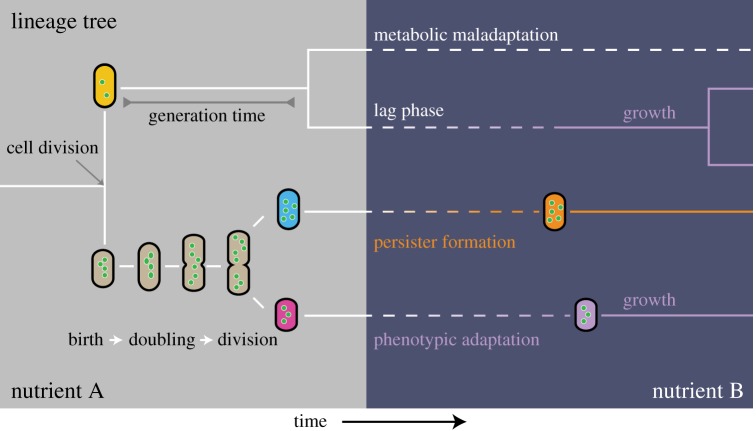
Conceptual framework for single-cell growth and phenotypic diversification upon a sudden nutrient transition in isogenic populations. During steady-state exponential growth of a population of isogenic cells, at constant environmental conditions, the total cell number increases exponentially. Individual cells progress asynchronously through their cell cycle. Cells vary in size, molecular composition and doubling time, due to inevitable stochastic effects, even those that are at the same cell-cycle progression and were born from the same mother [4,35,36]. When individual cells are suddenly confronted with a nutrient transition, not all of them have the capacity to adapt, even though they do have the metabolic machinery to grow on the new carbon source. This can lead to lag phases, temporary growth arrest [39] or maladapted states [7], and even the formation of persister cells in bacteria [11,40,41].

Real-time imaging of growth and fluorescent reporter-protein expression by single cells, with fluorescence microscopy [34], has drastically changed the way we think about populations of isogenic microbial cells (figure 1) [2,5,37]. These populations turn out to be inhomogeneous, with cells behaving as ‘individuals’. The molecular states of cells vary, in a dynamic, spontaneously fluctuating manner [4,35,36] (this aspect has been reviewed earlier [37,42]). A recent insight is that populations of isogenic cells can diversify into subpopulations with distinct phenotypes [5]. Such an adaptation strategy can be analysed in terms of a framework for fitness in dynamic environments [12,20]. A diversifying response may either be an evolved strategy or purely result from molecular noise, causing variation in cellular responses. In this section, we will discuss some striking examples that are provided by nutrient transition studies (figure 1).

### 3.1. A chance event can determine whether a cell adapts

A surprising finding was made with the lactose operon in *E. coli* [43], a system which was thought to be well understood. Whether a single cell initiates growth on lactose turns out to depend purely on a chance event in its recent past. This insight was gained by tagging the lactose permease with a fluorescent protein and tracking its expression in single cells. At intermediate induction of the lac operon a colony consists of two different phenotypes: cells with high and low expression [44]. A cell has to reach a threshold permease level in order to commit to lactose growth. Only when the expression level is high enough, a positive feedback mechanism becomes active that enhances permease expression to a level required for growth. This expression threshold has to occur before the cell is aware of lactose in its environment, because it lacks sensors for it and lactose cannot pass the membrane by diffusion. At intermediate induction, the lac repressor dissociates randomly from the lac promoter and occasionally leads to a burst of transcription activity that, if it lasts long enough, can lead to the threshold level expression of permease, priming the cell for lactose growth when it is present. As a result of this, the response times of *E. coli* cells to a sudden lactose addition are very broadly distributed, because it can take a long time before cells reach the threshold expression level of the permease [10]. Chance therefore decides when cells adapt. This is an example of stochastic adaptation. Evolution simulations indicate that bistability of the lac operon may not be so prominent in natural settings [45].

### 3.2. Responsive adaptation leads to more homogeneous responses of all cells

When cells perceive the extracellular environmental change, e.g. via a dedicated sensor, cells can respond much more homogeneously. This is illustrated by a study with the budding yeast, *Saccharomyces cerevisiae*, in which the number of transcripts of the gene *MET5* was counted in single cells. *MET5* is required for the synthesis of methionine, when it is absent from the environment [46]. By changing the sulfur source in the medium from methionine to sulfate, the dynamics of *MET5* induction could be monitored. It was observed that individual cells exhibited nearly identical response times. Although there were still differences in adaptation times (i.e. the time needed to induce gene expression) between individual cells, all cells eventually adapted. The spread in adaptation times is mostly a consequence of transcriptional noise and much less due to differences in the timing of perception. Clearly, cells perceived the presence and absence of methionine with high precision. The entire population shifts uniformly to the new state within a relatively short time period (compared to the generation time). The presence of an initial variability in transcription activity is expected to have only a minor influence on cellular fitness.

### 3.3. The phenotypic state of a cell can cause it to maladapt

Examples exist that indicate that a subpopulation of cells is not able to initiate growth on a new carbon source, or one that is suddenly increased in concentration. When yeast cells are, for instance, exposed to a glucose transition, a small fraction arrests growth, because they were in a deviating metabolic state [7]. Different metabolic states are most probably caused by varying enzyme concentrations and can result in depletion of cellular adenosine triphosphate (ATP) when the rate of upper glycolysis exceeds the rate of lower glycolysis by too much. Similar behaviour is observed with *E. coli* cells [11,40,41], although this behaviour probably originates from a different molecular mechanism.

### 3.4. Distinguishing generalist from specialist adaptation strategies

When discussing different phenotypes, we usually distinguish subpopulations that vary greatly in growth rate, e.g. growing versus non-growing [7,11,40,41]. The situation can also be more subtle. A nice example exists where different phenotypes show varying capacities for growth [47]. In this study, yeast cells were exposed to alternating levels of glucose and maltose. Fluorescent labelling of an enzyme required for using maltose, combined with time-lapse microscopy allowed the tracking of different phenotypes. It was shown that the phenotypes that initiated growth on maltose grew slower when they were switched back to glucose, compared to the phenotype that never performed the switch to maltose. This means that adapting to a new environment may depend on the cell's history. Different wild yeast strains displayed differences in lag time after the switch [47,48]. It was proposed that this is due to different levels of catabolite repression and that two different strategies could be identified. A ‘specialist’ strain has high levels of catabolite repression, which gives it a growth rate advantage on a specific nutrient, while a generalist grows slower on specific substrates, but switches faster and achieves higher growth rates on other substrates.

### 3.5. Cell density-dependent subpopulation formation

**Figure 2.**
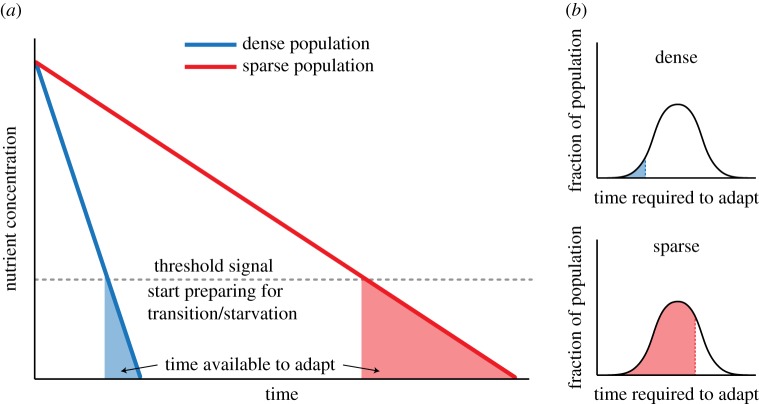
The ability of individual cells to successfully transition from one nutrient state to another is dependent on population density. (*a*) The rate at which a nutrient such as glucose disappears from the environment is determined by the cell density. Shown are two scenarios, where a very dense (blue line) and a very sparse (red line) population consume a nutrient. In both cases, low nutrient levels are detected at some threshold concentration, at which point cells have to prepare for nutrient depletion. (*b*) The time needed to prepare for a new condition will differ for individual cells (this can be described by a distribution), depending on their exact state at the time a threshold signal is detected. If the nutrient abruptly runs out, as in the case of a very dense population, only a small fraction of cells will be prepared for the new condition. When the population density is low, the time window will be large and most cells will adapt in time.

Solopova *et al.* [39] grew *Lactococcus lactis* in the presence of two different carbon sources, glucose and cellobiose. Glucose is the preferred carbon source. Cells sense when the glucose concentration drops below some threshold (figure 2*a*) and initiate gene expression to prepare them for growth on alternative substrates, such as cellobiose. What Solopova *et al.* [39] found was that cell density determined the fraction of cells that successfully make the transition from glucose to cellobiose growth. Specifically, the higher the cell density, the lower the fraction of cells that resume growth on cellobiose. The explanation for this finding probably lies in the time required for individual cells to prepare for a transition from a substrate like glucose to another, such as cellobiose. Implementing the physiological changes required for growth on cellobiose (e.g. expression of new metabolic genes) takes time, and any differences between individual cells, at the moment that low glucose is sensed, will result in some cells needing more or less time than others to prepare (figure 2*b*). The time available to all cells is determined by how quickly the remaining glucose disappears from the environment. At high cell densities, this will happen very quickly and only a small fraction of cells will manage to make the necessary changes for growth on cellobiose before glucose is depleted; cells that fail to do so will be stuck in a physiological state that is incompatible with cellobiose consumption. If, on the other hand, cell densities are low when the threshold is sensed, the rate at which glucose disappears will be slow and most cells will have sufficient time to prepare for the new condition.

## 4. Chance events that can impact the fate of a cell

**Figure 3.**
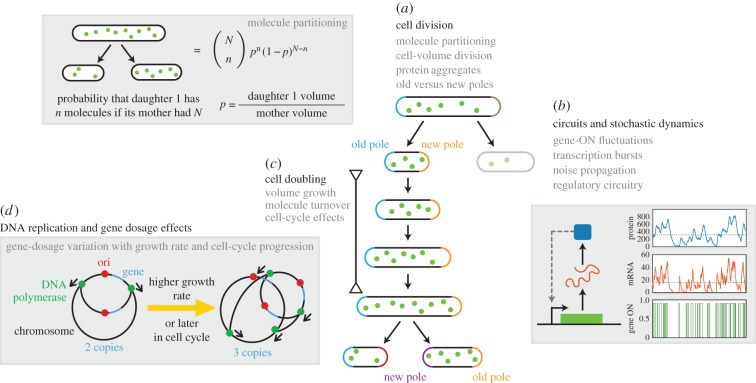
Overview of molecular and cellular stochastic processes. Numerous mechanisms contribute to the cell-to-cell variability of cellular characteristics during steady-state, exponential growth. (*a*) Molecule partitioning can be described by a simple model based on the ratio of daughter and mother volumes. Protein aggregation at the poles can lead to distinctive protein concentrations in daughter cells and determines pole age. (*b*) Gene expression consists of multiple stochastic processes. Transcription occurs in bursts and this noise can propagate to the protein level. Network motifs can further modulate the effect of noise. (*c*) Cell doubling, dilution by growth and cell cycle progression influence the physiological state of the cell. (*d*) The position of the gene on the chromosome and the number of chromosomes determine the level of gene dosage effects.

The previous examples of phenotypic adaptation indicate that chance events co-determine the fate of single cells upon an environmental change. The types of chance events found so far can be categorized into four classes: (i) molecular origins, (ii) cellular and systemic effects, (iii) cell-cycle stage and DNA-replication dependencies and (iv) history effects (figure 3).

### 4.1. Molecular stochasticity

Biochemical reactions are inherently stochastic, which leads to fluctuations in concentrations of macromolecules [49]. At large numbers of reactant molecules, the behaviour of biochemical reactions is predictable, because fluctuations in copy numbers are negligible (box 1). Stochasticity in molecule copy numbers can make systems inherently non-deterministic when those numbers are low. Transcription factors and mRNAs are typically present at such low numbers. The lifetimes of these molecules determine the rate and duration of fluctuations [50,51]. While fast fluctuations typically average out during one cell cycle, slow fluctuations that exist on time scales equal to or longer than the cell cycle can provide a ‘molecular memory’ [52–55]. The size of a fluctuation in the copy number of a molecule is set by the size of the imbalance between the synthesis and degradation rate of a molecule, and how quickly the system dissipates the fluctuation [49]. The fluctuation size can be quantified as the relative width (dispersion) of the probability distribution that describes the copy number of a molecule in each cell and is called noise. Noise can propagate in networks [56], and get amplified or attenuated along the way, leading to systemic phenotypic variations in populations of clonal cells. In box 1, we briefly summarize the great variety of molecule stochasticity effects that have been discovered.

**Box 1. Molecular stochasticity.**

*Quantification of stochasticity: the noise measure* Noise measures the magnitude of cell-to-cell variability. For an isogenic population, it quantifies the dispersion (relative width) of the distribution of measured cellular characteristics as var/*m*^2^ with var as the variance and *m* as the mean of those measured values. As variances of independent events are additive, variance is used rather than standard deviation. To get an idea: a system with constant synthesis and first-order degradation, i.e. 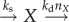, gives as steady-state noise for the number of protein *X*, *n*_*X*_: *m*/var^2^ = 1/*m* = *k*_d_/*k*_s_, indicating that noise is high when molecule copy numbers are low. The noise measure we refer to in the text is defined at a particular moment in time (called static noise).

*Transcription stochasticity and bursts* The copy numbers of transcription factors, genes and mRNA are generally low in microbial cells; transcription stochasticity is therefore significant. As mRNAs are synthesized when the promoter is in its ‘ON state’, two time scales exist in mRNA dynamics; waiting times for consecutive mRNA synthesis events and for OFF to ON transition events. When this time-scale separation is pronounced, the gene is defined as ‘bursty’: then during the ON state mRNA is produced and degraded and during the OFF state it is only degraded, leading to much greater noise than when mRNA would be produced at a constant rate.

*Promoter design* Promoter design influences noise in mRNA and protein numbers [57–61]. Different designs affect the sizes and frequencies of transcription bursts due to, for instance, fluctuations in transcription factor (un)binding and DNA looping [62,63]. In yeast, the precise sequence and structural properties of the TATA-box influence noise [57,59,64].

*Chromatin effects in higher eukaryotes* As chromatin reorganization is a slow step involved in gene activity switching, tightly packed regions of the chromosome exhibit more noisy expression [65]. Genes under the control of nucleosome-free promoters are expected to exhibit lower (Poissonian) noise [66,67].

*Noise in protein copy numbers and network design* The extent to which fluctuations in mRNA levels are propagated to protein levels, and thus potentially lead to phenotypic diversification, is largely determined by mRNA translation efficiency and the ribosome-binding site [68] or particular mRNA codons [69]. While fluctuations in mRNA levels are typically fast, and average out during the cell cycle, fluctuations in protein levels are slower and can persist over several generations [35]. The number of transcription binding sites in the promoter region also affects noise [60,70]. The noise in transcription factor numbers can propagate to the expression of their target genes [56]. Regulatory motifs, for example, a negative feedback, can attenuate noise or shift it to different components of the regulatory network [71–74]. The noise characteristics of a gene sometimes reflect the dynamics of its modulators [75].

### 4.2. Chance events at cell division

In symmetrically dividing bacteria, such as *E. coli* and *Bacillus subtilis*, division produces, on average, two equally sized new-born daughter cells. While mechanisms exist that ensure the equal partitioning of DNA content [76], other molecules are partitioned in a more random manner such as via random diffusion in the cytosol, or based on cellular localization (like membrane or pole proteins [77,78]).

Freely diffusing molecules are inherited in approximately equal concentrations by the daughters, even if they differ in birth volumes, provided their copy numbers are high. The noise (variance divided by the mean squared) in the number of proteins that a daughter with birth volume *V*_1_ received from its mother, with *N* molecules and volume *V*, equals (1/*N*)((*V*/*V*_1_) − 1), which quickly becomes negligible for large *N*. Differences in the size of the two daughters will lead to differences in their absolute molecule numbers, which can diversify their behaviour.

An interesting example of non-diffusive partitioning is that of RNA polymerase (RNAP). Bakshi *et al.* [79] showed that ≈82% of RNAP is bound to DNA; the remainder concerns two pools, which are in equilibrium with the bound pool. RNAP abundance therefore correlates strongly with DNA content (and not with cell volume). Volume differences between two daughter cells will therefore result in RNAP concentration differences, with higher concentrations of RNAP in the smallest daughter. Yang *et al.* [80] showed that cell-to-cell variability in the RNAP concentration leads to heterogeneous protein expression across cells.

The partitioning of molecules during division, be it through active or passive processes, nearly always has a chance component. For molecules present at low numbers, partitioning errors can be large in the absence of mechanisms that coordinate segregation [77,78]. Eukaryotic cells, like yeasts, contain a variety of specialized organelles, including mitochondria, vacuoles and lysosomes, that are present at much lower numbers than most proteins and metabolites. Cells can partition those low abundant structures more evenly, using dedicated actin-dependent transport processes [81,82]. Even if segregation involves such coordinated mechanisms, Huh & Paulsson [83] showed that accurate partitioning of this class of low abundant organelles and molecules is extremely difficult to achieve and that cell-to-cell variability is almost inevitable. This conclusion is experimentally supported by the finding that cell-to-cell variability in mitochondrial content probably arises from errors in partitioning during cell division [84].

Asymmetric partitioning of molecules or organelles also has a biological function. For example, yeast differentially enriches particular proteins in mother and daughter cells, using an active sorting mechanism [85]; a process that has been linked to ageing in these cells. Mother cells age because they retain damaged, or lifespan limiting, proteins such that their daughters start with a younger, ‘reset’ physiology. Levy *et al.* [86] found that replicative ageing influences cell-to-cell variability in protein expression: the abundance of a protein involved in trehalose biosynthesis, TSL1, correlated with the number of divisions a cell has undergone and that this, in turn, correlated with its survival chance upon sudden heat stress. Others have shown age-related asymmetry in mitochondrial function [87]. These findings suggest a general functional role of asymmetry in partitioning as a means of rejuvenation, but more importantly underscores the fact that cells of different chronological ages differ physiologically.

Age-related asymmetric protein partitioning has also been described in *E. coli* [88–91]. *Escherichia coli* cells are rod-shaped, with two poles. Upon division, each daughter cell receives an old and a new pole. A cell's age can be quantified by the number of times its old pole has been inherited. In the case of *E. coli*, cell age correlates with growth rate and older cells appear to accumulate protein aggregates [88–90]. Beyond growth rate effects, the consequences of age-dependent protein aggregation in bacteria remain unclear, but the finding that in *Mycobacterium smegmatis* cell age and antibiotic susceptibility correlate (albeit weakly) [92] suggests a possible role in physiological heterogeneity.

### 4.3. DNA replication and gene dosage

Symmetrically dividing cells double their molecular content from birth to division at steady-state, exponential growth (i.e. balanced growth). While the abundance of most molecules increases in proportion with cell volume, gene copy number (i.e. gene dosage) is an exception, due to the discrete nature of DNA replication [93]. As cells generally grow asynchronously—some have just been born, while others are halfway through their cell cycle or are about to divide—DNA content and gene copy number vary between cells at different stages of the cell cycle. This can cause cell-to-cell variability in their molecular constitution if the production rates of proteins are sensitive to changes in gene dosage [94].

In bacteria, the circular genome is copied in a bi-directional linear manner; starting from the origin of replication (oriC) towards the terminus (terC). This is a more or less continuous process [94], although periods without active replication are observed under slow growth. DNA replication leads to a sudden discrete increase in gene copy number. In bacteria, this change in gene dosage propagates to protein content [93,94], and the chromosomal position of a gene determines the concentration dynamics of the associated protein [94]. In eukaryotes, where replication occurs exclusively during the S-phase of the cell cycle, gene dosage-dependent effects appear to be suppressed by some mechanism, probably involving chromatin modifications [93].

### 4.4. Cell-cycle effects in eukaryotes

The eukaryotic cell cycle comprises distinct stages, consisting of growth (G1 and G2), DNA synthesis (S), mitosis and cytokinesis (M). Cell-cycle progression is achieved by a complex protein network that imposes checkpoints to ensure orderly transitions from one stage to the next. In *S. cerevisiae*, studies have shown that progression through these stages is accompanied by global rearrangements in almost all cellular processes and that structured cell cycle-dependent changes occur across many layers of organization, including the transcriptome [95–97] and the metabolome [98,99], as well as in protein localization [100] and organelle morphology [100].

Distinct cell-cycle stages (growth, DNA synthesis, mitosis and cytokinesis), at which metabolism is different [9], enhance the cell-to-cell variability in an asynchronously growing population of cells. Individual cells will be in different physiological states according to their position in the cell cycle. In yeast, cell cycle-dependent gene-expression variation exceeds variability due to stochastic fluctuations in gene expression, even for noisy promoters [101].

### 4.5. Molecular memory and history effects

Cell-to-cell variability is not only influenced by spontaneous fluctuations inside a cell but also by its history, including that of its (immediate) ancestors [35,55]. The molecular composition of a newborn cell is determined by that of its mother at division.

The inheritance of molecules from their ancestor cell gives microorganisms a ‘molecular memory’ that can confer a fitness advantage [24,43,55]. Once again, the *lac* operon in *E. coli* has proved to be an excellent model to demonstrate this effect. *Escherichia coli* cells that passed the expression threshold of *lac* permease (*lacY*) in the past were more likely to commit to phenotype switching upon reinduction after several generations of growth in the absence of *lacY* induction [24,43]. In the absence of an inducer, no new *lacY* proteins are produced; the existing proteins dilute by volume growth and are partitioned into daughter cells. Owing to the long lifetime of *lacY* , in comparison with the generation time, the *lacY* levels decrease only slowly over several generations. Cells that have been repeatedly induced will commit faster to growth on lactose than cells whose ancestors did not express *lacY* in their recent history.

A similar history dependence has been observed during cell fate decisions by *B. subtilis*. Cells are primed for differentiation to a new phenotypic state several generations before they actually commit to it [102,103]. Alternatively, they can maintain a phenotypic state for a predetermined period of time [54].

The phenomenon of molecular memory is not limited to bacteria. Recent studies indicate that the ability of yeast cells to respond to nutrient changes depends on past nutrient availability, several generations earlier. This was found during repeated switches between glucose and galactose [104]. The underlying mechanism involved the inheritance of cytoplasmic proteins and particular chromatin modifications [104].

With this universal mechanism of ‘passive transmission’ of stable molecules from mother to daughter in mind, it is not difficult to envisage that cell-cycle and environment-independent (long-term) oscillations in gene expression or metabolism will have an effect on phenotypic heterogeneity [9,105–107]. While these oscillations can introduce synchrony among cells of an extant population [105,106], cells born in different phases of the oscillations will show variations in their molecular make-up and probably react differently to environmental cues due to their distinct phenotypic state.

## 5. Persister cells: a case study for the fitness consequences of chance events

The previous sections considered examples of phenotypic diversification upon changes in nutrient availability. In the context of nutrient shifts, it is often not clear whether subpopulations emerge as part of a fitness-enhancing strategy or whether a fraction of the population maladapts. While theoretical arguments are often offered to support claims that phenotypic heterogeneity improves the adaptive flexibility (which is implied to be fitness-enhancing) of cell populations, experimental demonstrations are limited in scope and difficult to generalize. In the paragraphs that follow, we consider the phenomenon of bacterial persistence, which is an example of non-genetic phenotypic heterogeneity that confers a fitness enhancement.

### 5.1. The role of stochasticity in persister cell formation

**Figure 4.**
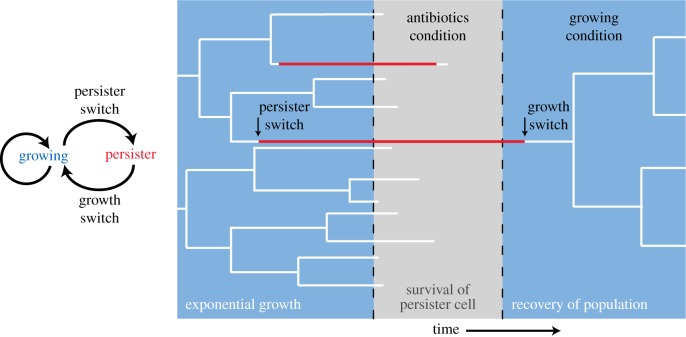
The fitness advantage of spontaneous formation of persister cells during steady-state exponential growth. Persister cells can form spontaneously under constant conditions of steady-state exponential growth, conferring a fitness advantage when a sudden extinction-threatening condition occurs, such as an antibiotic [6,108]. Normally growing cells, or persisters that switch back to a growing state, will probably succumb to the effects of the antibiotic.

Isogenic populations of bacteria can contain subpopulations of cells that are slow-growing and generally highly tolerant to antibiotics and other stresses (figure 4); these cells are called persisters [109,110]. Their formation appears to be a survival mechanism that protects the population from extinction, when sudden harsh conditions occur. Most often, but not exclusively, these antibiotic tolerant subpopulations are formed via a mechanism called stochastic phenotype switching [6,108]. This is a spontaneous process that occurs even during exponential growth and is a prime example of stochastic adaptation [5]. Owing to continuous switching, a subpopulation of persister cells is always maintained. Persisters can, however, also be formed via responsive adaptation; i.e. in response to particular environmental conditions such as stresses [111], nutrient transitions [11,40,41] and at the onset of the stationary phase, when at least one nutrient is depleted [112].

That persister cells are slow-growing does not fully explain why they are less susceptible to antibiotics, not even to antibiotics that directly influence growth processes, because a faster growth rate can sometimes also reduce susceptibility [113]. Persistence is therefore explained by a distinct physiological state [41]. The physiology of persistent cells, hence their degree of tolerance and the duration of their persistent state, is dependent on whether they have been formed during a nutrient transition, removal or depletion [114]. For example, persister cells formed during a nutrient transition show a different response to antibiotics from that of tolerant cells formed upon a removal of a nutrient [41]. The size of these persister subpopulations can vary greatly with conditions [6,11,40,41,108], ranging from about 1 in a million under conditions of fast, steady-state exponential growth [6], to almost the entire population switching to this state in response to certain nutrient transitions [11]. While the molecular details of the mechanisms behind persister formation differ, stochasticity plays a central role.

### 5.2. Stochasticity and persister formation during steady-state exponential growth

The role of stochasticity in persister formation is best understood during steady-state, exponential growth [6,108]. The ‘alarmone’ molecule (p)ppGpp plays a central role herein. It is a key control molecule in *E. coli* that tunes the bacterium's physiology as a function of growth rate [115]. When it is high in concentration, (p)ppGpp inhibits growth processes and activates general stress and stationary-phase systems. At fast exponential growth, i.e. on rich or mineral media supplemented with glucose, the concentration of (p)ppGpp is generally low. However, when (p)ppGpp fluctuations occur in a cell under these conditions, concentrations can spontaneously rise to a threshold level and induce a non-growing state. A self-perpetuating positive feedback mechanism then ‘locks’ this cell in the persister state, via the activation of toxin/antitoxin (TA) systems [108,116]. In this manner, growing and non-growing cells can stably coexist in an environment that supports fast growth. Thus, at exponential growth, cells switch into the persister phenotype by chance; their conversion back is also probably a chance event, but this is less well understood.

TA systems play a central role in the spontaneous formation of persisters during exponential growth and in the adaptive response to stress conditions. The design of TA systems allows for the coexistence of a non-growing fraction in an exponentially growing population. The HipA toxin and HipB antitoxin, for example, are both constitutively expressed, generally in a balanced manner. When the ratio of HipA toxin to HipB antitoxin exceeds a certain threshold, be it through a random or regulatory event, persistent cells are formed [117]. Evolutionary tuning of transcriptional regulation, protein stability or TA interactions [118] could therefore affect the probability of whether a particular cell exceeds the threshold. In turn, changes in the fraction of persisters and their lifetimes can be fitness-increasing or -decreasing, depending on the environmental dynamics. As a consequence, the probability for spontaneous persister formation itself is expected to be evolvable. In fact, antibiotic-resistant strains are known to sometimes carry *hipAB* mutations [119], and experiments have indeed shown that evolution can shape system properties to match the antibiotic treatment pattern [120].

### 5.3. Stochasticity and persister formation during nutrient transitions

Nutrient transitions can lead to the appearance of phenotypically distinct subpopulations [7,11,39] (see §5.2), often distinguishable as growing and non- (or slow) growing fractions. Heinemann and colleagues [11,41] found that non-growing subpopulations of *E. coli*, which appear after transitions from glucose to gluconeogenic substrates (e.g. acetate or fumarate), exhibited increased antibiotic tolerance with characteristics similar to the persister phenotype. They found elevated levels of (p)ppGpp and activation of TA systems upon a nutrient shift, indicating that the mechanisms of persister formation during exponential growth and upon nutrient transitions are related. Tolerant cells formed upon nutrient removal are, however, more susceptible to an antibiotic treatment than those formed during a nutrient transition, as their low catabolic capacity cannot match the ATP-maintenance requirements. They do have in common that the RpoS-mediated generalized stress response is activated, probably triggered by elevated levels of (p)ppGpp. Radzikowski *et al.* [41] concluded that persister formation is induced by a strong deviation from metabolic homoeostasis upon a change in nutrient availability, such that synthesis of new proteins required for new catabolic activity cannot be realized. Instead, cells take a less ‘risky’ strategy and invest into stress response and cellular maintenance, which is apparently less costly and leaves the metabolic state of the cells intact. The metabolic origin of this persistence state was underscored by demonstrating that persister cells quickly resuscitated upon re-addition of glucose.

### 5.4. How persister formation relates to a trade-off between instantaneous and long-term fitness

An increase in the heterogeneity of key molecules in stress response systems (e.g. TA system) can improve single-cell fitness and as a consequence, population survival. Even though several aspects of the cellular stress response indicate that it is optimized for speed, such as the constitutive expression of TA systems to ensure rapid growth arrest when needed, there is still an adaptation time. Sudden extinction-threatening conditions are therefore probably beyond the capacity of responsive systems. Here, the continuous formation of persister cells, via stochastic, reversible phenotype switching provides a solution, as a subpopulation of stress-tolerant cells renders the population always prepared for sudden adverse conditions. However, this readiness comes with a trade-off. It reduces the instantaneous fitness of the population; the non- or slow-growing subpopulation of persister cells will only make a small contribution to the generation of new cells. Evolution therefore plays an important role here: via tuning of the switching kinetics, this trade-off can be optimized [15,17,23,24,121,122].

### 5.5. Other examples of phenotypic heterogeneity with fitness consequences

Persister subpopulation formation provides an example of how molecular stochasticity can be fitness-enhancing. A major challenge in single-cell studies is to figure out whether, and under which conditions, phenotypic heterogeneity has fitness consequences. Clearly demonstrating that it impacts fitness is not trivial. A few other examples exist, however, that show how heterogeneity affects a population's adaptive capacity. These examples include the ability to invade a new niche, better preparation for changes in a current niche and the ability to deal with extinction-threatening conditions.

A clear model that illustrates how noisy expression can expand or open up new niches was given by Ackermann *et al.* [123]. They showed how the heterogeneous expression of virulence factors by *Salmonella typhimurium* leads to two distinct subpopulations: one that ultimately sacrifices itself so that the other can invade a new environment. In this scenario, a phenomenon called self-destructive cooperation involves a small phenotypically distinct subpopulation that, through expression of a particular virulence factor, can trigger an inflammatory response in the gut. This results in both the elimination of intestinal microflora and the subpopulation itself, but in doing so removes competitors and allows the remaining *S. typhimurium* population to invade.

An example of phenotypic heterogeneity leading to better preparedness in changing environments is provided by the finding that the galactose regulatory pathway is activated in a fraction of the cell population of *S. cerevisiae*, hours before glucose is fully consumed [124]. This strategy reflects the trade-off between the cost of being prepared, in terms of growth rate and unnecessary enzyme expression, and the ability to make a fast switch as a population. In contrast with the previous example, this does not necessarily involve any change of niche. However, once there is competition for resources with other species within the same niche, making a fast switch has a clear fitness advantage.

Heterogeneous gene expression can also affect fitness negatively, as demonstrated by Deris *et al.* [125] in a study on antibiotic-resistant *E. coli* strains. They show how molecular fluctuations underlie a bistability, via global feedback between growth and gene expression, producing subpopulations with reduced expression of proteins that protect against antibiotic action. In this case, an isogenic population of antibiotic-resistant individuals diversify into growing (resistant) and non-growing (sensitive) subpopulations for a range of drug concentrations. This is clearly an example where chance leads to phenotypic heterogeneity that reduces, rather than enhances, the fitness of a population.

Most probably the best understood example of how molecular noise can improve cellular fitness by improving the functioning of a molecular circuit is bacterial chemotaxis. This is one of the few systems in molecular biology for which we have a mechanistic understanding of its functional systemic properties (i.e. tumble bias and adaptation time) and we can study how these properties affect *E. coli*'s fitness [18,126]. The functional properties of the chemotactic circuit are very sensitive to the concentration of its proteins, which fluctuate. For instance, the tumble bias is a determinant of the distance that *E. coli* travels and protein fluctuations cause isogenic cells to vary in their travelled distances [18]. This cell-varying foraging behaviour is advantageous when conditions change, e.g. from a steep to a shallow nutrient gradient. Noise in tumble bias then ensures that some cells search for food in small areas, while others cover longer distances. In this case, phenotypic heterogeneity enhances the fitness of the genotype.

That fluctuations in phenotypic behaviour can be fitness-enhancing and evolvable is illustrated by Beaumont *et al.* [127]. They showed that subjecting *Pseudomonas fluorescens* to a switching environment led to the evolution of a bet-hedging genotype, which switched randomly between phenotypes.

## 6. Emerging concepts in the study of phenotypic adaptation

We have discussed examples of adaptation strategies of microorganisms when they are confronted with particular environmental dynamics. We emphasized the fitness effects of chance events. Some species adapt purely by chance, via stochastic phenotypic diversification [5], in order to prepare for future conditions. In this strategy, the size and lifetimes of the resulting subpopulations co-determine fitness [15,17,19]. Adaptation is more deterministic when sensing–response mechanisms are used. In these strategies, phenotypic diversification is undesired. Noise still plays a role in the variability in the magnitude and timing of the response [128]. Which adaptation strategy is best can be decided through an analysis of its fitness effects, in experiments [16,23,24] and theory [12,13,18,129,130]. We consider this an important research direction that may ultimately lead to a comprehensive theory of microbial phenotypic adaptation mechanisms and strategies.

The evaluation of the fitness of a particular adaptation strategy involves at least two descriptions. One is systemic, and ideally involves measuring the one-dimensional fitness value, by challenging a microbial population with specific changing conditions [6,7,16,23,24,39]. The other is the characterization of the molecular mechanisms for adaptation, including the stochasticity of its dynamics in single cells, to assess cell-to-cell variability in adaptivity, including maladaptation and phenotypic diversification [7,16,108]. With theory and experimental studies of carefully chosen mutant strains, the fitness effects of the strategy can be assessed.

Figuring out the fitness contribution of stochasticity to an adaptation strategy is a complex problem. It dates back to earlier studies in population genetics [131] and several innovative studies have recently been carried out that address this using a combination of experiments and theory [16,23,24,39,132]. One complicating aspect is that stochasticity is an inevitable consequence of molecular and cellular processes [35,49] and that it is therefore often questionable to what extent its magnitude has evolved [133]. And, even if stochasticity is fitness contributing, this will probably be so only in particular environments, and we generally do not know the evolutionary history of microorganisms. Exploratory theory and simulations [12,15,17,19,121] can then help in sharpening our thoughts and intuitions, and suggest informative experiments that could further reveal the surprisingly diverse roles of stochasticity in microbial fitness [6,7,16,23,24,39].

Several constraints that shape phenotypic adaptation by microorganisms have recently been identified. Firstly, the allocation of finite biosynthetic resources has proved to be an important limit that constrains the protein expression profile of microorganisms [134–136]. The molecular circuits in a cell that are responsible for different tasks, such as catabolism, anabolism and stress systems, compete for limited biosynthetic and cellular resources, such as transcription and translation machinery, cytoplasmic and membrane space [21,22]. As many reaction rates depend linearly on enzyme concentrations, higher enzyme concentrations are generally advantageous for cellular processes, enforcing resource competition [137]. A second emerging constraint is that cells turn out to have limited phenotypic plasticity and sensing capabilities. Cells simply do not have all the circuits required for growth and survival in particular environments encoded on their genome; they may simply lack certain metabolic pathways. Nonetheless, the metabolic plasticity of some microorganisms is truly amazing. *Escherichia coli*, for instance, is expected to grow on hundreds of carbon sources [138]. Even though it has this latent capacity, it lacks sensors for the majority of the nutrients it can in principle grow on; i.e. *E. coli* does not have hundreds of carbon source sensors [139]. This could partially explain why *E. coli* shows a limited capacity to restore growth when carbon sources are suddenly changed, where some cells fail to initiate growth [11]. On the other hand, some cells are capable of switching, indicating that metabolism does have the capacity to restore growth on new carbon sources, but this is dependent on a cell state-dependent mechanism that is partially understood [41]. Limited membrane capacity [22] and the reduction in growth rate, when proteins are produced that are not directly needed [135], may drive microorganisms towards reducing their sensing capacities. Finally, many constraints exist that prevent a cell from tracking its environment. Molecular noise in sensing, transcription and translation circuitry is an obvious effect that causes isogenic cells to grow differently in the same environment [140]. Another reason why perfect environmental tracking is impossible is that protein synthesis is costly [135]. In short-lived environmental states, the benefits from newly expressed proteins cannot be reaped to recover the investment costs of biosynthetic resources, leading to a net fitness loss [24]. The lag time, associated with cellular responses, limits the number of offspring cells can make during a transition to a new condition, especially when it is of a short duration [24]. Sometimes responding fast, or even anticipating some environmental transitions [5], can have big fitness advantages.

## 7. Concluding remarks

During the last decade, it has become clear that non-genetic heterogeneity pervades all aspects of biology. It has prompted a re-evaluation of the way we think about many cellular phenomena, including our views on microbial fitness and adaptation. In this review, we discussed many mechanisms that are now known, or thought, to generate non-genetic variability. However, their biological role is not always fully understood. For example, despite numerous studies on molecular and cellular stochasticity (both experimental and theoretical), only a handful have managed to demonstrate clear effects on fitness. It is often unclear how much of the observed cell-to-cell molecular variability serves a biological function, and how much of it simply reflects the robustness (or lack thereof) of the underlying molecular circuits. Related to this unknown is an important practical question: to what extent can these cellular stochastic phenomena be artificially manipulated? While non-genetic phenotypic heterogeneity is fascinating from an evolutionary perspective, randomness and unpredictability are often undesirable in biotechnological or biomedical settings, where they can significantly impact culture performance or treatment efficacies. If it is simply biochemical limitations that cause much of the variability we observe, then it may be difficult to override or steer. On the other hand, if a noise source has been tuned by selective pressures, it seems reasonable to expect that engineered alterations are possible. In this regard, theoretical approaches, combined with synthetic molecular biology, will be pivotal in untangling the complex relationship that exists between stochastic molecular and cellular processes and the phenotypic characteristics of individual cells.

## Data accessibility

This article has no additional data.
